# Influence of resin cement shade on the color and translucency of zirconia crowns 

**DOI:** 10.4317/jced.56425

**Published:** 2020-03-01

**Authors:** Ghada Ayash, Essam Osman, Lucette Segaan, Mohmmad Rayyan, Christelle Joukhadar

**Affiliations:** 1 Department of Oral Rehabilitation Sciences, Faculty of Dentistry, Beirut Arab University; 2 Clinical Instructor Department of Oral Rehabilitation Sciences, Faculty of Dentistry, Beirut Arab University

## Abstract

**Background:**

Zirconia crowns are highly attractive for clinicians, although have poor translucency when used as single restorations, in addition to unknown effect of resin cement shade on final cemented crown shade. This study aimed to assess effect of resin cement opacity on color replication potential of different zirconia frameworks with target tooth color, in addition to different zirconia crowns translucency evaluation.

**Material and Methods:**

Twenty-four zirconia crown restorations were fabricated to restore single central maxillary incisor for 8 patients, divided into 3 groups according to color and type of zirconia used (white Zr core, colored Zr core and monolithic HT Zrcowns). Each group was further subdivided into 2 subgroups according to resin cement shade. Using Easyshade spectrophotometer, Delta E color difference was calculated between each crown parameters using 2 different resin luting cement shades and adjacent target tooth. Translucency parameters (TP) were tested for finished crowns. ΔEs obtained were assessed based on ΔEof 1.6 which represented color difference that could not be detected by human eye and considered clinically acceptable.

**Results:**

No statistically significant values were found between subgroups related to different resin cement shade. Translucency parameters showed statistically significant different values. Monolithic crowns showed highest translucency parameters followed by Zr crowns on white cores then Zr crowns on colored cores.

**Conclusions:**

Resin cement shade didn’t affect final color perception. Monolithic high translucency crowns usage gained advantages of high translucency and delamination prevention. Zirconia crowns could be cemented by opaque or transparent cement without affecting final color.

** Key words:**Zirconia, resin cement, monolithic, translucency, spectrophotometer, Delta E.

## Introduction

Color matching of restorations with adjacent natural teeth remains challenging in esthetic dentistry (1). All ceramic restorations have higher translucency; thus, used in esthetic region. Brittleness was considered limiting factor, during initial utilization of all-ceramic restorations, consequently improvements and enforcement modifications in these restorations have substantially enhanced their resistance. Current high-strength all ceramic systems using aluminum oxide and zirconia based frameworks exhibit very good clinical data and high attractiveness (2).

Three factors could alter final color of crown in color matching of all ceramic crowns; color of prepared tooth, color of crown and color of luting cement, therefore to reproduce natural tooth color, these factors have to be optically harmonized (3). Earlier studies recommended that, in order to mask influence of underlying discolored tooth, or abutment color on final restoration color, ceramic thickness should be at least 2mm. In the other hand, in vital tooth, 2mm axial reduction remains challenging without pulpal violation and weakening remaining tooth structure durability. Hence, using cement with suiTable color might be best existing solution to mask substructure color and its effect on final color restoration, since it is not practical to accomplish ideal ceramic thickness (4).

Although, effect of resin cement color on ceramic restorations final color has been previously evaluated (5); however, no clinical suggestion is available regarding usage of different cement colors in clinical situation. Consequently, upon introduction of new ceramics with different translucencies, more studies are needed to understand substructure outcome and cement color on ceramic final color.

Instrumental measurements of translucent restorative materials using spectrophotometer, colorimeter, spectroradiometer and digital camera plus software have been reported (6). Spectrophotometers are among most accurate tools for color change (ΔE) (7). ΔE value is often used to compare two different colors and calculated using following equation: ΔE= (ΔL2 + Δa2+ Δb2) ½.

Study hypotheses were; there is no difference in translucency of different zirconia based crowns and resin cement opacity has an effect on resultant shade.

## Material and Methods

-Specimen grouping 

Twenty-four ceramic crowns were fabricated for 8 patients recruited for their need to restore single ceramic crown on maxillary central incisor, each patient received 3 different zirconia crowns. This sample size was sufficient for statistical evaluation and following Consort 2010 sample size determination. Patients were fully informed about clinical study purposes, design and consents were obtained prior to treatment. Study design was accepted by IRB committee at BAU (2014H-003-D-P-0012).

The selection criteria were that; all patients should have at least one of adjacent teeth sound to serve as guideline and control for shade determination, small proximal fillings that would not affect buccal aesthetic appearance were accepted and good oral hygiene and awareness. All patients were randomly divided according to color and type of Zr crowns into 3 categories and divided into groups according to shade of resin cement used (each type of Zr crowns was tried first with transparent resin cement and then with A3 shaded resin cement).

-Initial shade recording

All teeth were cleansed and polished with plain white dentifrice, using low speed rubber-polishing cup. Intraoral spectrophotometer (EasyshadeV,Vita, Yorda Linda, North America) was used to record preoperative shades instrumentally for central incisor to be restored and target adjacent tooth as control shade. Each tooth underwent spectrophotometer analysis at circular areas of 1.5mm on middle of labial surface with probe tip positioned at 90 degrees to tooth-surface (8). Color qualification of standardized circular area was based on CEI-Lab system and was expressed according to L*, a* and b* color parameters (9). Data were recorded 3 consecutive times. Following another calibration cycle, entire process was repeated for adjacent tooth.

-Teeth preparation for crown restoration

Teeth preparation were done according to silicone index fabricated for each patient to aid in controlling tooth reduction amount incisally and axially. 0.9 mm subgingival heavy chamfer finish line was prepared with 1.2mm axial reduction and 1.8 mm incisal clearance using round end tapered guided pin diamond burs (10). Preparations finishing were performed to ensure smooth surfaces by guided pin finishing burs.

-Interim restoration

Using preformed silicone index made for fabrication of provisional restoration, interim composite resin material was placed in it then over prepared tooth surface until setting, removed, adjusted and cemented with eugenol free temporary dental cement.

-Final impressions and crowns fabrication 

Final impressions were made in another session to give proper time for marginal periodontium healing and give time for interim restoration to mold gingiva to prevent bleeding during retraction cord placement (11) using double cord for gingival displacement. After pouring in type IV stone and preparing accurate dies, they were scanned using Cercon eye scanner. Zirconia copings and monolithic crowns were produced by milling zirconia blanks and were sintered according to manufacturer instructions. For groups WC, 0.5 mm white zirconia core was milled from Cercon base light blanks. For groups CC, 0.5mm colored zirconia core was milled from Cercon base colored blanks.

**Figure 1:**
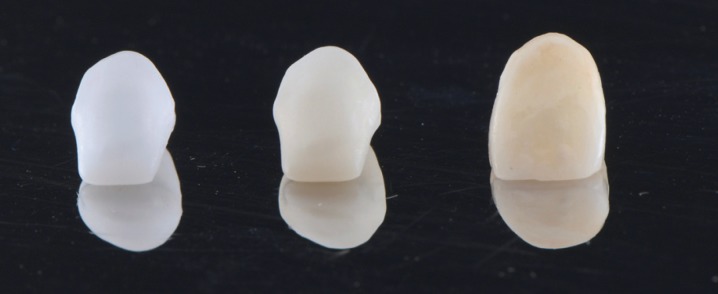
Color measurement of colored Zr core on white background and color measurement of white Zr core on black background.

**Figure 2:**
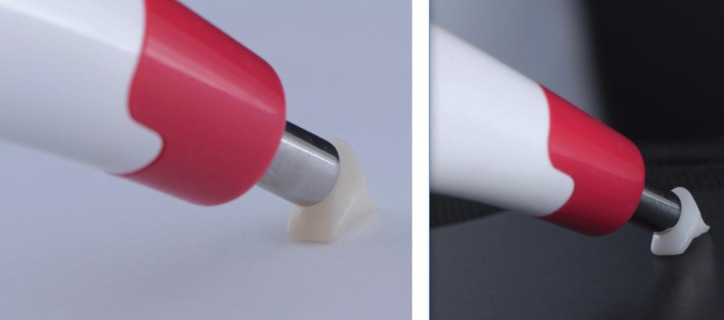
White Zr core, colored Zr core and ht Zr crowns.

For group MZ, full anatomical monolithic high translucent zirconia crowns were milled from Cercon HT blank by type 0 Cercon brain expert cutters usage and sintered at 1.500οin heat furnance (Fig. 1). Manual finishing after sintered was done for all groups to both inner and outer surfaces of copings using air particle abrasion with 100 µ alumina particles. Each coping and crown were trial fitted to its corresponding tooth. Cleaning was accomplished by aid of steam cleaner. Easyshade V was used for recording color coordinates (L,a,b) of each coping/crown (Fig. 2). Copings were cemented using silicone index for group WC and CC whereas for group MZ, body stains were used as external stains on crowns.

-Try in and cementation 

Completed crowns consecutively tired on corresponding patient for proper fit, contacts and occlusal adjustments (if needed) before they were sent for glazing.

All intaglio surfaces were air particle abraded using 100µm Al2O3 then zirconia primer was applied. Proper cementation procedures were accomplished by resin cement usage (Multilink Automix, IvoclarVivadent AG, Liechtenstein).

-Determination of color and translucency

Translucency is property of substance that permits passage of light, but also disperses light, so objects cannot be seen through material. Porcelain translucency is usually measured with translucency parameters (TP) or contrast ratio (CR) (6). CR is defined as ration of illumination (Y) of tested material when placed over a black background (Yb) to illuminance of same material when it is placed over white background (Yw). TP is defined as color difference (ΔE) between uniform thickness of material over a white and black backing (12).

 In current study, color of crowns was measured, before and after resin cement placement of shades A3 and transparent respectively, according to CIELABsystem, with standard illumination D65 in reflectance in spectrophotometer, operating in length range of light spectrum λ=360-740 nm. Color of each experimental group was measured in 3 coordinate dimensions of L*[ from 0 [black] to 100 [white], a* green-red (-a*=green; +a*=red) and b* blue-yellow (-b*-=blue; +b*=yellow). ΔE value was calculated according to: ΔE=[(L*1-L*0)2+(b*1-b*0)2]1/2.

In which subscripted 0 represents measured color in reflectance mode of ceramic without cement (control) and subscripted 1 represents ceramic color with respectively underlying cement. Translucency parameters (TP) of each crown was determined by calculating difference in color between readings over black (standard calibration tile with CIE L*=24.58, a*=0.27, b*3.57L) backgrounds. TP was calculated according to: TP==[(L*B-L*W)2+(a*B-a*W)2+(b*B-b*W)2]½, which subscripts B and W refer to measurements made on black and white backgrounds.

TP is defined as color difference of material of given thickness over white and black backgrounds and corresponds directly to regular visual assessments, TP value of zero corresponds to completely opaque material and greater TP value means higher actual translucency of material.

CIE color coordinates L*, a*,b* were calculated in each of 3 areas of ceramic crowns. Areas of interest were measured 3x3 mm in cervical, body and incisal region. Triplet measurements were performed and average readings were used for data calculation. Color data obtained from adjacent target teeth were considered as control. Color difference ΔEbetween control and zirconia crowns color was calculated as follows.

• ΔL*= L*control- L* experimental

• Δa*= a*control- a* experimental

• Δb*= b*control- b* experimental

• ΔE*= (ΔL *2+Δa*2 +Δb *2)1/2

-Statistical analysis

Results were recorded, tabulated and statistically analyzed. Numerical data were explored for normality by checking distribution of data, calculating mean and median values as well as using tests of normality (Kolmogorov- Smirnov and Shapiro-Wilk tests). Color parameters (L*) and (b*) data showed parametric distribution while (a*) parameter, color change (ΔE) data showed non-parametric distribution. Data were presented as mean, standard deviation (SD), minimum, maximum and 95% confidence interval (95% CI) values.

For parametric data (L*and b*); repeated measures ANOVA test was used for comparisons between different groups and control group, to study effect of core type on color parameters and to study effect of cement on color parameters. Bonferroni’s post post-hoc test was used for pair-wise comparisons when ANOVA test is significant.

## Results

Results of current study were divided into 4 main parts: effect of cement shade on color parameters, effect of cement presence on color parameters, effect of cement shade on color difference and translucency parameters (TP).

-Effect of cement shade on color parameters (Table 1).

While using core + veneer + A3 cement as well as core + veneer + Transparent cement; there was no statistically significant difference between the three core types for all the 3 parameters L*, a* and b*.

**Table 1:**
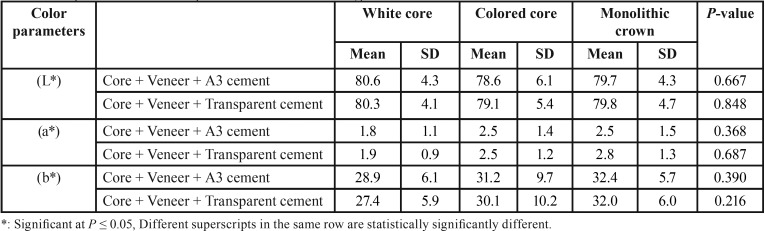
Comparison between color parameters of different core types.

-Effect of cement presence on color parameters

As regards (L*) parameter of the crowns with different core types; there was no statistically significant difference between (L*) values with and without cement (Table 2). Similarly, for (a*) parameter of crowns with all core types; there was no statistically significant difference between (a*) values with and without cement (Table 2). While for (b*) parameter of Gr I; A3 cement showed the statistically significantly highest mean (b*) value (28.9 ± 6.1). There was no statistically significant difference between without cement and transparent cement; both showed statistically significant lower mean values. As regards Gr II as well as Gr III; there was no statistically significant difference between (b*) values with and without cement (Table 2).

**Table 2:**
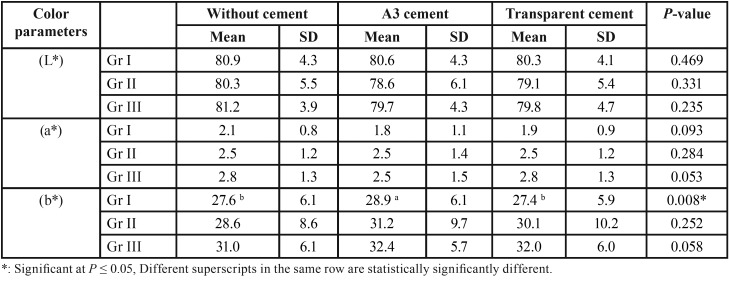
Comparison between color parameters of different groups with and without different cement shades.

-Effect of cement shade on color difference (ΔE)

Either with white, colored or monolithic core types; there was no statistically significant difference between color changes (ΔE) values with A3 cement, transparent cement, as well as without cement (Table 3).

**Table 3:**
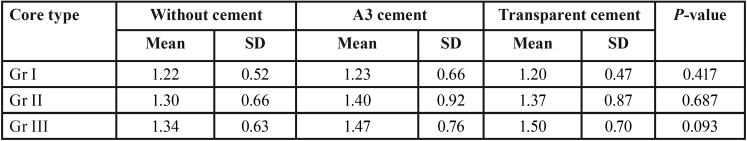
Comparison between color changes (ΔE) with and without cements.

-Translucency parameter (TP)

•Descriptive statistics

Descriptive statistics of Translucency Parameter (TP) are presented in Table 4. Gr III recorded highest mean of TP (22.25 ± 4.73) followed by Gr I (20.87 ± 3.55). Gr II recorded the lowest mean TP value (16.59 ± 4.37).

**Table 4:**
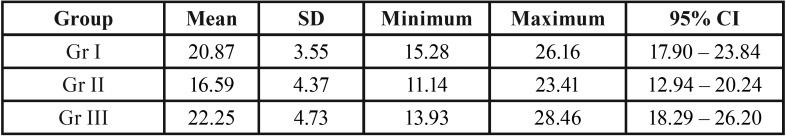
Descriptive statistics of Translucency Parameter (TP) for the different groups.

•Effect of core type on Translucency Parameter (TP)

**Figure 3:**
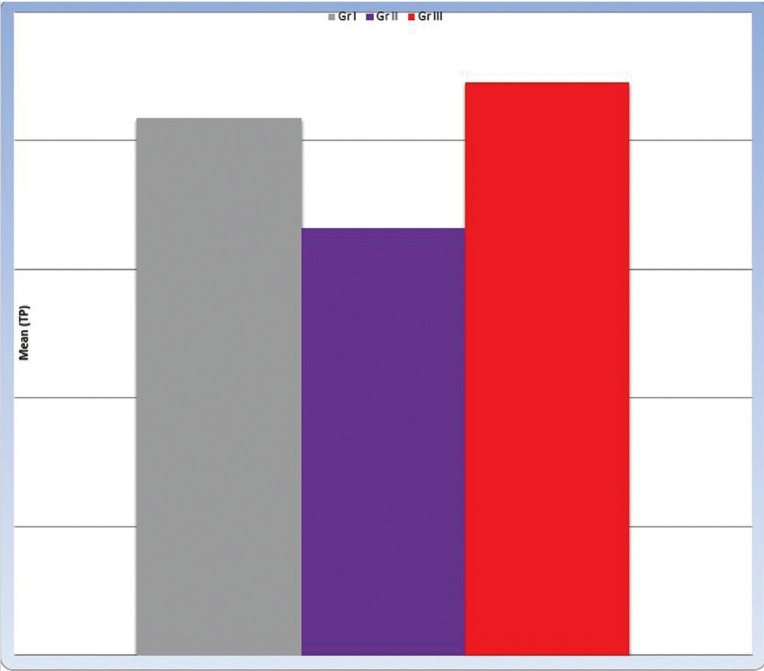
Bar chart representing mean (TP) values with different core types.

The P-value between Groups I, II and III was statistically significant (0.044*). There was no statistically significant difference between Gr I (20.87a± 3.55) and Gr III (22.25a± 4.73); both showed the statistically significant highest mean (TP) values in comparison with Gr II (16.59b± 4.37) which showed the statistically significant lowest mean (TP) (Fig. 3).

## Discussion

Replication of natural teeth is challenging, especially with single-tooth restorations. Due to individuality of each single human tooth, it is great order to create single anterior restorations that match harmoniously to existing adjacent teeth (13). Demands for highly esthetic restorations with advances in prosthetic fabrication processes and techniques have led to introduction of zirconia crowns (14).

Achievement of all ceramic esthetic restoration that matches perfectly with adjacent teeth is interplay result between 2 important optical factors: on one hand, masking capacity of ceramics to block background color (in many cases, non vital dentin or core buildup material) with sufficient material thickness or opaque masking liner and on the other hand, amount of translucency of ceramic that will allow natural appearance. Different zirconia substructures have been suggested to improve esthetic results of zirconia ceramic crowns (15). However, final color results is unpredicTable when restoration is compared of different layers with unspecified thickness, which is the case for core veneers all- ceramic restorations (16). In present study, Cercon CAD CAM system was used for its ease of scanning and milling steps and availability of different white, colored and translucency blanks with superior esthetics.

Benchmark instrument used was Easyshade Vspectrophotometer in both “tooth mode” for recording natural tooth color and “restoration mode” for recording ceramic color. Significant advantages with spectrophotometric measurements include ability to analyze principal components of series of spectra and ability to convert data to various color measuring systems (17).

Resin cement was used in current study, because of its higher strength, ability to reduce fracture of ceramic materials and low solubility in oral fluids (18). It is true that Zr crowns could be cemented using glass ionomer cements (19), but due to high GIC solubility and slowness reaching maximum strength , they are not preferred for cementation as they may cause crown fracture.

Spectrophotometric color measurements in terms of CIE L*, a*, b* color coordinates for different Zr cores were prepared, Color differences value (ΔE) represented numerical distance between 2 colors each having coordinates L*, a*, b* (20). Purpose of present study, was to test effect resin cement shade on final shade of zirconia based crowns milled from different blank shades to match adjacent teeth shade and to calculate color difference between restoration and natural control tooth. Translucency parameters measurements were also studied.

Translucency parameters were measured and found statistically significant between 3 tested categories. Highest mean was for TP values of MZ (22.25) followed by WC (20.87) then CC (<16.59). Current study was in agreement with study done by Tuncel *et al.* 2013, in which they evaluated effect of shade of coloring liquid on translucency of zirconia framework and effect of coloring liquids should be taken into consideration during fabrication of restorations with zirconia framework (21). In MZ, highest mean value was attributed to high translucency blanks used. Veneering procedure didn’t play a role because it was standardized between groups. Moreover, effect of difference in ceramic thickness didn’t show up.

In current study since both white and colored cores were milled with same design and thickness; 3 different zirconia crowns had same shape due to index use and even the same perceived visual color by help of technician’s skills. Translucency parameters were calculated with zirconia crowns placed on black and white backgrounds. These readings were recorded after addition of veneering ceramics to stimulate clinical conditions accurately from one side and to be standardized with 3 different zirconia frameworks because monolithic zirconia was directly milled into full contour has no core alone from beginning to be tested. TP calculations were recorded instead of CR since Y illuminance was not available after Easyshade readings.

Heffernan *et al.* in 2002 compared translucency of 6 all-ceramic materials veneered and glazed at clinically appropriate thicknesses and found that glazing cycle resulted in decreased opacity for all test materials except completely opaque In-Ceram Zirconia and metal-ceramic specimens (22). In present study, for standardized purposes and to neglect effect of glazing cycle, glazing procedure was similar for 3 tested groups.

Baldissara *et al.* in 2010 reported different translucency values for different zirconia materials with direct transmission method and light flow instead of contrast ratio. They reported that all materials evaluated may be considered translucent to a certain degree, although quantity of transmitted light is not remarkable when compared to value of positive control flow (23). This support current definition of zirconia as “semi translucent” core material. It should be taken into consideration that translucency measurements for zirconia cores are carried out on core material in absence of veneering material and not representative of usual clinical conditions, in which presence of veneering porcelain can reduce translucency, as function of thickness, opacity and of veneering material itself (24,25). In contrast, Chang *et al.* in 2009 found that final color of translucent cores was influenced by color of cement and it was possible that this could affect zirconia core as well. Fazi *et al.* in 2006, also found influence of opacity of cement on color of thon Lava zirconia frames. This was attributed to Lava material which is characterized by its translucency in comparison to other zirconia material (26).

In current study, 2 resin cement shades were selected, A3 and transparent shade. Results showed that there was no significant variation observed among mean CIE L*, a*, b* color parameters of various specimen combinations tested regarding color of cementing medium used. This result was in accordance with Azer *et al.* in 2006, who tested effect of esthetic core shades and different shades of resin cements used and found that there was not statistically significant final color difference through effect of altering cement shade (27). Results were not in agreement with Vichi *et al.* 2000, who tested influence of ceramic and cement thickness on masking of various types of opaque posts and found that final color of all ceramic crowns with 2mm thickness did not show significantly appreciable color difference for final esthetics with various underlying substrate, resin cement and thickness (28). Nevertheless, when ceramic thickness decreased to 1.5mm, it was advised to tale substrate aspects into consideration and esthetic corrections might be done mildly with different cement shades. Authors used IPS-Empress glass-ceramic which is more translucent than zirconia and might be reason for different results with thin ceramic thicknesses.

Strong point worth mentioning in current study was that, most of papers related to color coordinates, specimens used were discs (29,30) whereas study crowns used stimulating appropriate clinical conditions. Surprisingly, color had no minimal effect on final shade of crowns. It implies necessity to purchase expensive colored blanks is no longer needed. Drawback of colored blanks on bond between zirconia core and veneer could be avoided.
